# What is the impact of distraction osteogenesis on the upper airway of hemifacial microsomia patient with obstructive sleep apnea: a case report

**DOI:** 10.1186/s40001-021-00547-1

**Published:** 2021-07-13

**Authors:** Rongyang Wang, Shixing Xu, Ruimei Yang

**Affiliations:** 1 Department of Stomatology, Qilu Children’s Hospital of Shandong University, No 23976, Jingshi Rd, Jinan, 250022 Shandong China; 2 Department of Craniomaxillofacial Surgery, Plastic Surgery Hospital, Chinese Academy of Medical Sciences, Peking Union Medical College, Beijing, China

**Keywords:** Hemifacial microsomia, Distraction osteogenesis, Obstructive sleep apnea, Computational fluid dynamics, Upper airway

## Abstract

**Background:**

Current research about hemifacial microsomia (HFM) patients after distraction osteogenesis (DO) most emphasize the morphologic changes. This case report shows the outcome of DO on the upper airway of a HFM patient with obstructive sleep apnea (OSA) based on the use of computational fluid dynamics (CFD).

**Case presentation:**

An 11-year-old boy was diagnosed as HFM with OSA, and underwent unilateral DO. Polysomnography and CT scans were performed before and 6 months after treatment. After DO, lowest blood oxygen saturation increased from 81% to 95% and apnea and hypopnea index decreased from 6.4 events/hour to 1.2 events/hour. The oropharynx and nasopharynx were obviously expanded. We observed apparently increased average pressure, decreased average velocity and pressure drop in all cross-sections, and largely decreased airflow resistance and maximum velocity entirely in the airway.

**Conclusions:**

The results suggest that DO might be effective for the treatment of OSA by expanding the upper airway and reducing the resistance of inspiration.

## Background

Hemifacial microsomia (HFM), also known as the first and second branchial arch syndrome or hemifacial hypoplasia, is mainly characterized by unilateral mandibular maldevelopment [1]. Because of the impaired development of the affected side, the mandible progressively shortens and narrows, leading to concomitant reduction of the pharyngeal airway in HFM patients.

Distraction osteogenesis (DO), which has several advantages of reducing trauma, initiating adaptive changes of the soft tissues to enable greater bone movement and so on [2], has become a recognized curative treatment for children with HFM. Structural stenosis is one important pathogenesis for pediatric obstructive sleep apnea (OSA) [3], so except considering the changes of the soft and hard tissues, the postoperative outcome of the upper airway should be taken into accounts too.

The pathophysiological process of OSA is greatly affected by the flow field inner the airway. The investigation and analysis of the airflow is helpful for us to further understand the connection between the anatomical structure and function of the upper airway. However, due to the lack of direct examination methods and uniform standards, the research on OSA and the related problems have been greatly limited. With the interdisciplinary development of medical imaging and biomechanical technique, computational fluid dynamics (CFD) has been applied to patients with OSA widely [4–7], aiming to provide theoretical basis for the pathogenesis research, clinical diagnosis, and treatment strategy of OSA. Based on the previous studies, CFD has been verified as an effective technique to exactly calculate the airflow parameters [8]. However, few research have applied CFD to estimate the variation of upper airway after unilateral DO in HFM patients at present. This report was aimed to show the outcome of DO on the upper airway of a HFM patient with OSA based on the use of CFD, which may help to explore the therapeutic mechanism of DO for OSA.

## Case presentation

In accordance with Pruzansky–Kaban classification [9, 10], a diagnosed grade IIa male HFM patient, who was accompanied with OSA and underwent unilateral DO for mandibular advancement was selected. He performed in-hospital overnight polysomnography (PSG) monitoring before starting DO and 6 months later. He had no tonsillar and adenoid hypertrophy, no previous orthodontic and orthognathic treatments.

Personalized surgical scheme was made according to the preoperative cephalometric and CT datasets. Surgical procedures such as extraoral submandibular incision, were carried out with the patient under general anesthesia. Distraction was started 7 days after operation, the frequency was 0.25 mm four times a day, the distance was 20 mm and the consolidation phase was 5 months. Finally, once the repeated CT scan demonstrated well osteogenesis, the distractor was taken out along the initial approach.

Based on the CT scans which were obtained before (T0) and 6 months after (T1) DO, image segmentation and smoothing of the upper airway was performed in Mimics 19.0 based on the threshold from −1024 Hounsfield Units (HU) to −259 HU not accompanied by paranasal sinuses. Subsequently, the model was converted into non-uniform rational B-splines surfaces in Geomagic Wrap 2017. Then the optimized model, which was saved as stereolithography format, was imported to Ansys ICEM CFD (Ansys 19.1, Canonsburg, PA, USA) to create an unstructured tetrahedral mesh.

**Fig. 1:**
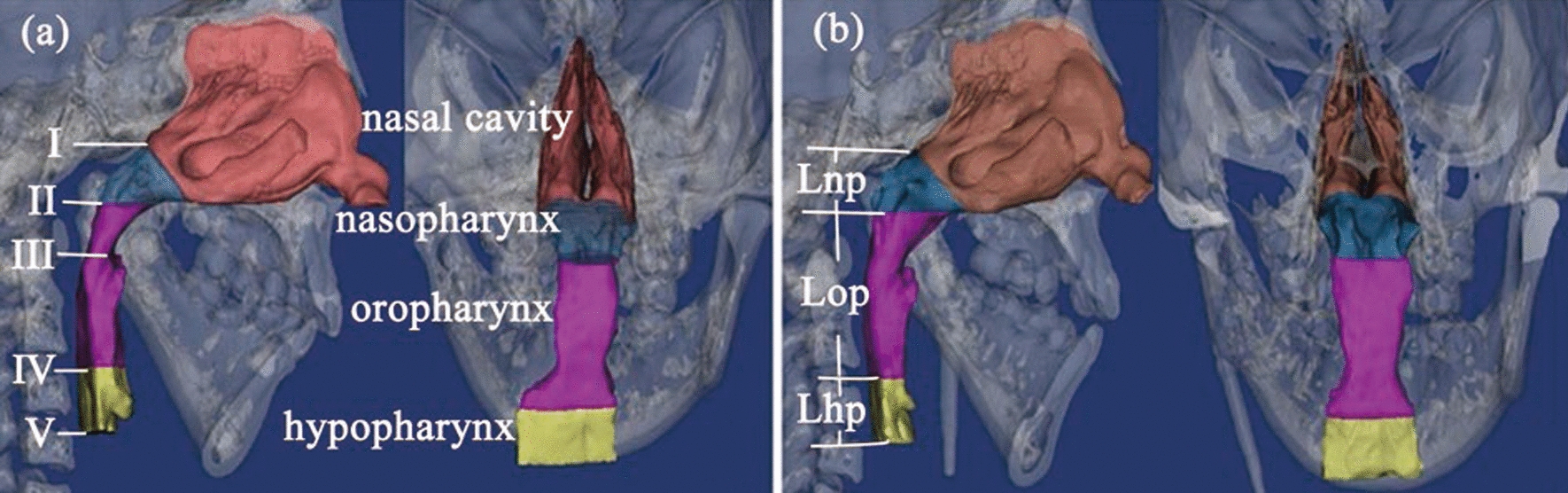
The reconstructed upper airway. **a** Pre-distraction: I—the choana plane, II—the plane parallel to Frankfort horizontal plane through PNS (posterior nasal spine), III—the minimum cross-sectional area plane, IV—the plane parallel to Frankfort horizontal plane through the superior border of the epiglottis, V—the plane parallel to Frankfort horizontal plane through the most anterior superior edge of the fourth cervical vertebra. **b** Post-distraction: Lnp—length of the nasopharynx, Lop—length of the oropharynx, Lhp—length of the hypopharynx

The transversal planes I, II, IV, and V, which traversed choana, PNS (posterior nasal spine), the superior border of the epiglottis and C4 (the most anterior superior edge of the fourth cervical vertebra), respectively, divided the upper airway into nasal cavity, nasopharynx, oropharynx, and hypopharynx, plane III was the minimum cross-sectional area plane (Fig. 1). Area of planes I–V, length of nasopharynx (Lnp), oropharynx (Lop), and hypopharynx (Lhp), and volume of each part of the airway were measured to evaluate the morphological variation.

Standard κ–ω turbulence model was used to imitate the airflow in a complete respiratory cycle with the nostrils as the inlet (impose a transient incompressible flow rate *Q* = 500 ml/s) and the bottom of hypopharynx as the outlet, the viscosity coefficient (1.789 × 10^−5^ kg/m/s) and density coefficient (1.225 kg/m^3^) were applied in the present research. Meanwhile, a non-slip boundary condition was adopted on the wall. The patient’s respiratory rate was 20 breaths per minute, so the respiratory cycle was 3 s. The second-order discrete scheme and SIMPLE scheme were applied to solve the pressure–velocity coupling. The average pressure, average velocity, the airflow pressure drop (Δ*P*) of cross-sections I–V, the maximum velocity (ν_max_) and Δ*P* of the nasal cavity, nasopharynx, oropharynx, and hypopharynx were computed at peak inspiration. The effective resistance (*R*) was computed by *R* = Δ*P*/*Q*.

### Follow-up

**Fig. 2:**
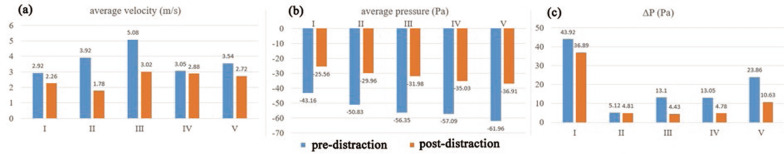
Comparison of the aerodynamic variables at the selected cross-sections (I–V). **a** Average velocity. **b** Average pressure. **c** Pressure drop (ΔP)

**Fig. 3:**
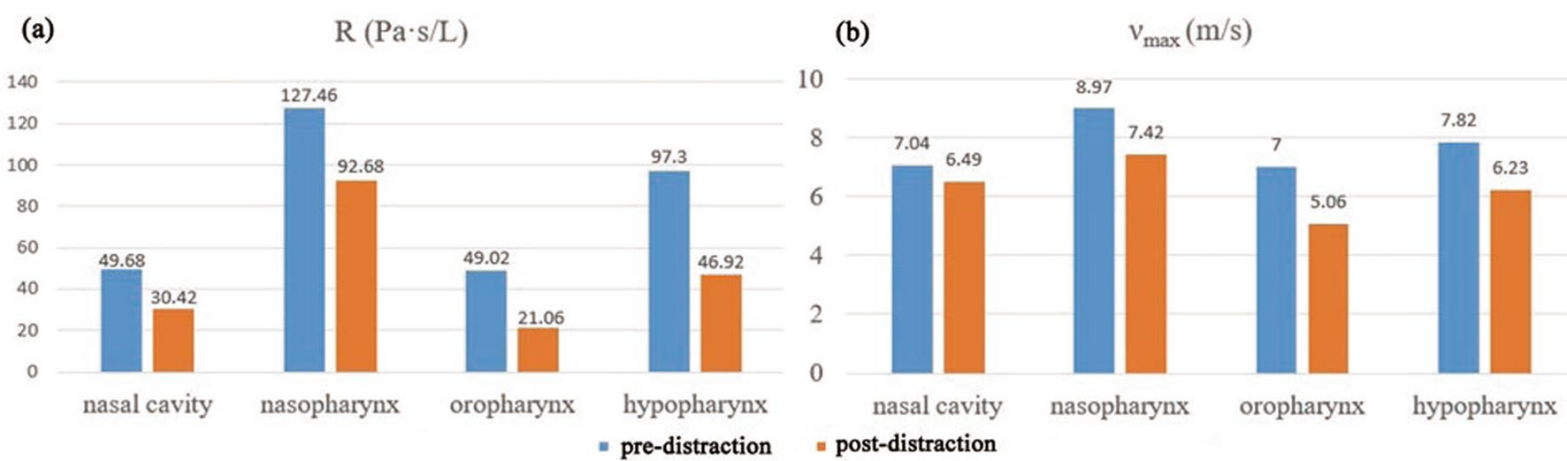
Comparison of R and ν_max_ in each part of the upper airway. **a** R: resistance. **b** ν_max_: maximum velocity

The patient’s clinical symptoms of OSA were significantly alleviated 6 months after unilateral distraction. The results of PSG examination before and 6 months after operation are shown in Table 1. The apnea–hypopnea index (AHI) was decreased and the lowest blood oxygen saturation (LSR) was increased. Morphological variations of the upper airway between pre-distraction and post-distraction are detailed in Table 2. Figure 2 displays the comparison of the average velocity, the average pressure and ΔP at the selected cross-sectional planes. Comparison of resistance and ν_max_ of the nasal cavity, nasopharynx, oropharynx, and hypopharynx is shown in Fig. 3.

**Table 1:** Comparison of the PSG tests before and after distraction osteogenesis

Variables	T0	T1
Age	11 years and 7 months	12 years and 1 month
BMI (kg/m^2^)	21.0	20.4
AHI (events/h)	6.4	1.2
LSR (%)	81	95

T0, before distraction osteogenesis; T1, after distraction osteogenesis

BMI, body mass index; AHI, apnea–hypopnea index; LSR, lowest blood oxygen saturation

**Table 2:** Comparison of the morphological variables before and after distraction osteogenesis

Variables	T0	T1	T1–T0	Change
Area (cm^2^)				
Plane I	2.77	3.04	0.27	9.75
Plane II	1.85	4.46	2.61	141.08
Plane III	1.38	1.95	0.57	41.30
Plane IV	2.49	2.17	−0.32	−12.85
Plane V	1.94	2.26	0.32	16.49
Length (cm)				
Lnp	1.36	1.38	0.02	1.47
Lop	3.55	4.01	0.46	12.96
Lhp	1.48	1.53	0.05	3.38
Volume (cm^3^)				
Nasal cavity	18.74	20.57	1.83	9.77
Nasopharynx	4.48	5.85	1.37	30.58
Oropharynx	6.13	9.13	3.00	48.94
Hypopharynx	3.70	3.57	−0.13	−3.51

T0, before distraction osteogenesis; T1, after distraction osteogenesis change: [(T1–T0)/T0] × 100%

## Discussion

Establishment of CFD models of the upper airway based on HFM patient with OSA to quantitatively estimate the changes of the airway after unilateral DO, might be helpful to explain the relationship between the airway morphology and function and improve the understanding of pathogenesis and treatment strategy of OSA. PSG tests were used to verify the effectiveness of CFD.

OSA is a disease with high incidence rate, many etiologies and complicated pathogenesis. Because of its characteristic repeated or incomplete obstruction of the upper airway and intermittent hypoxemia that occur during sleep [11], OSA has been considered as one of the important risk factors for hypertension, angina pectoris and cerebral vascular embolism [12]. Katz et al. [13] used PSG results to show that the duration of apnea–hypopnea > 2 breathing cycles, AHI ≥ 5 times/h, and LSR < 92% were the diagnostic criteria for children with OSA. In terms of treatment, surgical removal of its anatomical stenosis is an important principle for the treatment of OSA [14].

Among the surgical treatments, tracheotomy is the earliest, but it is only used in palliative or emergency situations. Uvulopalatopharyngoplasty only relieves the problem of soft tissue obstruction around the upper airway, and has no obvious effect on posterior lingual stenosis caused by mandibular retrusion, and the total effective rate is less than 50% [15]. Mandibular DO can make the mandible move forward and increase the tension of soft tissues such as mandible hyoid muscle, genioglossus muscle, and genioglossus muscle, so as to make the tongue root move forward, which can expand the upper airway, and fundamentally relieve the stenosis of the upper airway. Mandibular DO has been shown to be effective in avoiding tracheostomy or achieving early decannulation in OSA patients with retrognathic mandible. Because of the traction of the surrounding soft tissues, the advancement of the conventional orthognathic surgery is limited, the potential risk of bone recurrence would be increased along with larger mandibular advancement. However, comparing with sagittal split ramus osteotomy (SSRO), DO had a high major complication rate [16], ranged from 0% to 21.4%, most were local wound infection or neurosensory disturbance [17]. It was encouraging that out of the previously reported cases, the large majority were resolved with antibiotics, time and steroids [18]. Other potential problems of DO were patient noncompliance and nonunion/malunion, which did not worsen the results of the treatment significantly [19].

At present, many studies have shown that pediatric OSA involves multiple occlusive planes of upper airway. Therefore, acquisition of more accurate morphological data of the upper airway is necessary to locate the stenosis sites. Major et al. [20] pointed out that the upper airway had complex three-dimensional (3D) geometric structures. Due to the influence of OSA, the shape of the upper airway would generally have a certain degree of variation, and some information would be lost when it was transformed into two-dimensional images. In this study, the 3D reconstruction could obtain more accurate morphology of the upper airway.

In this case, the area of planes II and III, and the volume of oropharynx and nasopharynx showed obvious increase after distraction. As shown in Fig. 1, we can see that the upper airway before treatment was narrowed in the sagittal and coronal directions, especially in oropharynx. After treatment, with the mandible moving forward, the nasopharynx and oropharynx were expanded in the sagittal direction; in the coronal direction, the narrowing area of the affected side was expanded, and from the back view, the shape of the upper airway was more symmetrical. The average velocity achieved the peak at the narrowest part of the oropharynx before DO. The airflow velocity would decrease while the diameter of the airway would increase because of their proportional relationship [21]. Therefore, the average velocity of planes II and III was apparently reduced and *ν*_max_ in oropharynx appeared maximum decrease after distraction.

**Fig. 4:**
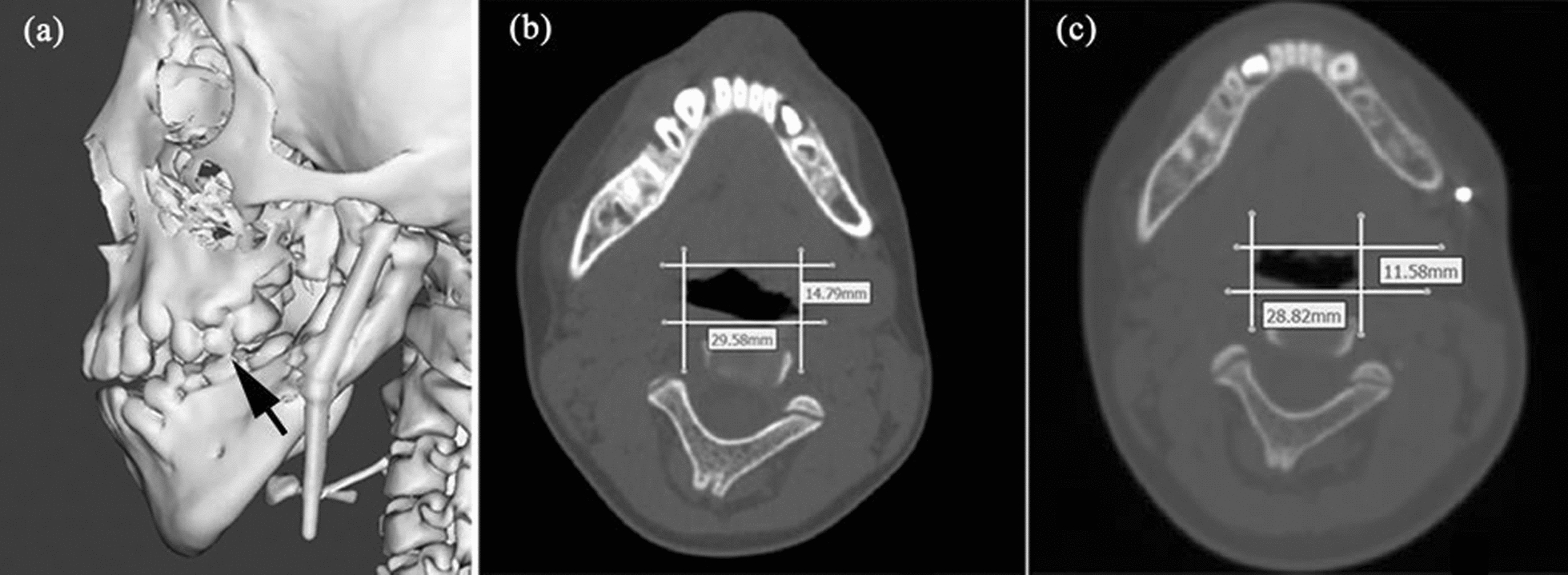
Comparison between the cross-sections at the superior border of epiglottis. **a** Post-distraction maxillofacial model indicating the premature contact of the left second primary molar. **b** Pre-distraction. **c** Post-distraction

After DO, the pharyngeal stenosis was greatly improved by skeletal expansion. Nevertheless, the area of plane IV and the volume of hypopharynx were slightly reduced unexpectedly. This is probably on account of the posterior shift of the tongue root, which might have resulted from the premature contact of the left second primary molar after DO and hypotonia of tongue muscles in the supine position during CT scanning (Fig. 4). However, we observed increased average pressure and decreased average velocity in plane IV, greatly decreased resistance and *ν*_max_ in hypopharynx, which were consistent with the performance of the expanded regions.

According to Bernoulli's principle, the pressure would increase when the airflow slowed down, which is consistent with our results of the CFD analysis. We observed greatly increased negative pressure and decreased ΔP in all selected planes and each part of the upper airway after distraction. Upon dilation of the stenosis by surgeries, airflow resistance usually decreases with reduction of the required pressure during inspiration. Similarly, the present case indicated that unilateral DO improved OSA by reducing resistance of the whole upper airway. The markedly improved AHI and LSR after distraction confirm the efficacy of unilateral DO on expanding the upper airway.

The establishment of the biomechanical upper airway models in OSA children can help us in better understanding the pathogenesis and evaluating the therapeutic effect of DO on OSA. In the past, the clinical diagnosis of OSA mainly depended on their clinical symptoms and PSG examination, but there was no better method to predict the actual stenosis site. The assessment of the morphology and internal flow field of the whole upper airway by computational modeling is helpful to make a more objective diagnosis of pediatric OSA. The 3D computer numerical simulation of this study was based on the established 3D accurate models of the upper airway according to the individualized CT datasets, and obtained the aerodynamic parameters by non-invasive CFD technology, and provided a theoretical basis for the evaluation of curative effects after DO.

## Conclusions

In this study, we established the CFD models of the upper airway of the HFM patient with OSA before and after unilateral DO to quantitatively evaluate the morphological and aerodynamical changes. Combined with the results of the preoperative and postoperative PSG monitoring, it was suggested that DO might be effective in the treatment of OSA by expanding the upper airway and reducing the resistance of inspiration.

## Data Availability

Not applicable.

## Abbreviations

HFM: Hemifacial microsomia
DO: Distraction osteogenesis
OSA: Obstructive sleep apnea
CFD: Computational fluid dynamics
CT: Computed tomography
PSG: Polysomnography
AHI: Apnea–hypopnea index
LSR: Lowest blood oxygen saturation
SSRO: Sagittal split ramus osteotomy
3D: Three dimensional
